# Effect of Pulsed Electric Field Pre-Treatment and the Freezing Methods on the Kinetics of the Freeze-Drying Process of Apple and Its Selected Physical Properties

**DOI:** 10.3390/foods11162407

**Published:** 2022-08-11

**Authors:** Dorota Nowak, Ewa Jakubczyk

**Affiliations:** Department of Food Engineering and Process Management, Institute of Food Sciences, Warsaw University of Life Sciences—SGGW, Nowoursynowska 159C, 02-776 Warsaw, Poland

**Keywords:** pulsed electric field, vacuum freezing, fast and slow freezing, freeze drying, colour, water activity, drying curves

## Abstract

The aim of this study was to investigate the effect of application of pulsed electric field (PEF) and different freezing methods (fast, slow and vacuum freezing) on the drying kinetics as well as selected physical properties of freeze-dried apple. The apples were subjected to PEF treatment with range of pulses from 0 to 160 and the intake energy from 0 to 1327 kJ·g^−1^. Apples with and without PEF treatment were frozen with different rates and the freeze-dried. The water content, water activity and colour attributes of freeze-dried apples were investigated. Regression analysis and fitting procedures showed that among six different models, the Midilli et al. model the best described the drying curves of all dried samples. The highest value of the parameter L* = 71.54 was obtained for freeze-dried sample prepared without PEF pre-treatment and fast frozen. Application of PEF pre-treatment resulted in increase in browning index of freeze-dried apples (*BI*). The studies confirmed the positive effect of PEF on the freeze drying rate only in the case of the slow or fast freezing of the material after the application of low-energy PEF treatment. However, the increase in drying rate was also observed after application of slow and vacuum freezing of the material without PEF pre-treatment. These technologies can be recommended for optimization of the freeze drying process of apples. The statement that the freeze drying process with application of appropriately selected PEF processing parameters causing only partial destruction of cell membranes can be considered as an innovative contribution to the development of science about the possibilities of PEF application.

## 1. Introduction

Freeze drying is a drying method that allows obtaining products with low water content and activity, high nutritional value, that are easy to reconstitute, and microbiologically safe [1,2,3]. However, evaporation of some volatile compounds, denaturation of some proteins, oxidation of bioactive compounds as a result of high porosity, phase transformations, and high hygroscopicity may occur during the freeze drying process as well as storage of products [1]. It is based on the phenomenon of ice sublimation. However, the product must be frozen. The production cycle includes: freezing, sublimation, desorption, and packaging [4]. Due to the initial freezing, all physiological processes and biochemical reactions are stopped, which allows to maintain the chemical composition almost identical to the raw material [4,5]. Additionally, the freeze-dried product retains the taste and aroma of the fresh product, which is especially important for almost all food raw materials, both of plant and animal origin. During the process, the product is either frozen or dry. The lack of free water prevents the mechanical deformation of the material (no material shrinkage or very little shrinkage), which allows the preservation of the internal structure and shape [6,7]. For example, volume changes in apple, melon, pear, and strawberry at 25 °C and 11% RH were in the range 1–10% [8]. The freeze-dried material has high porosity [8] and low water content, which causes crispness, crunchiness, and brittleness of products [3,9] as well the hardness decreased very much with the drying process [9,10].

During sublimation, the temperature of the material is negative, and the vast majority of nutrients are not degraded [1,11]. Evaporation at negative temperature reduces the loss of volatile substances with water vapor, which preserves the aromatic and taste compounds. Freeze-drying allows retention of the colour of the dried product. The rehydration capacity of freeze-dried products is very high [5]. All these features make freeze-dried food the closest food to fresh food, and a very attractive product on the food market.

During the freeze drying, mass transport and heat conduction are diffusive. Thus, this resistance can be reduced by controlling the size of material, in practice, it comes down to a suitable reduction in material size [12]. In the case of food raw materials with a cellular structure, a significant resistance to heat and mass transport is the presence of cell membranes [3]. Due to their semi-permeable nature, cell membranes limit the transport of water vapor. These results caused an increase in the freeze-drying time. Therefore, an important issue related to the reduction in the freeze drying time is the disintegration of cell membranes [4]. It can be obtained by thermal denaturation or by slow freezing [13]. Blanching may have a negative effect on the texture of the product and result in the loss of sensitive nutrients, aromas and dyes [13]. On the other hand, during slow freezing, large ice crystals are formed, which mechanically damage delicate cell membranes [14]. This is due to the volumetric expansion of water during the transition from liquid to solid [15]. Both of these methods are of little use in practice. Therefore, treatment with Pulsed Electric Field (PEF) is a promising technological method in food preservation [16,17] and onto different industries, including the pharmaceutical industry [18]. For example, it was stated, that PEF treatment at 25 kV cm^−1^ could improve the quality of grapefruit juice [19]. Ribas-Agustí et al. [20] observed that PEF processing could change the apple functional value, by either increasing phenolic contents or the phenolic bioaccessibility. Pulsed electric field (PEF) pre-treatment of apple tissue was linked to higher crystallinity of the freeze-dried samples, which is important during storage. Additionally, the higher water absorption of dried apples with PEF application (better reconstitution properties) was observed in comparison to untreated samples [21].

The Pulsed Electric Field (PEF) method uses electric fields to induce plasmolysis of cells (electroplasmolysis). The application of PEF permanently or temporarily disrupts the integrity of cell membranes, increasing their permeability, although the mechanism of action of this technology has not been fully understood [22]. Electroporation occurs because the cytoplasm and intercellular fluid have a greater dielectric constant than the cell membrane. The difference between these constants results in a transmembrane potential of about 1V which induces electrical breakdown of the membrane. This is called critical tension [23].

Critical values of the applied electric field for plants and microorganisms are in the range of 1–2 and 10–14 kV·cm^−1^, respectively (e.g., *Escherichia coli*) [24]. The key parameters for PEF are the value of the electric field *E* (kV·cm^−1^), the temperature of the medium (°C), frequency of the applied electric field (Hz), number of pulses (*n*), pulse width (μs) and process time (s) [15,25].

Freeze drying requires the material to be frozen. For this, air or contact freezing can be used. Recently, studies have been undertaken on the possibility of using vacuum freezing [26]. Vacuum freezing is the result of the rapid evaporation of water from the material under reduced pressure. Vacuum freezing is very fast and can be used in liquids due to the lack of water diffusion resistance (e.g., freezing instant coffee before freeze-drying) [27].

Destruction of cell membranes, which are resistant to water diffusion with PEF, increases the intensity of evaporation [28], which, theoretically, could freeze the material at the stage of pressure reduction in the lyophiliser.

The series of recent studies has indicated the possibility of using a PEF pre-treatment to allow vacuum-freezing operation as a first period of the freeze drying process (as method of freezing the material before sublimation) [21,29]. PEF-treatment significantly affected the decrease in sample temperature below zero until −12 °C for apple tissue with a degree of degradation of 0.96 [21].

Vacuum-frozen and freeze-dried apple disc-shaped samples (29 mm in diameter and 5 mm in thickness) were characterised by the reduction in the freeze drying time which was positively correlated with the amount of energy applied during PEF treatment [30]. Lammerskitten et al. [21] observed that PEF pre-treatment of apples reduced the freeze drying time to moisture ratio of 0.004 by 57%. On the other hand, the freeze drying time required to obtain a higher *MR* equal to approx. 0.01 (approx. 8.5 w.b.) for apples after and without PEF treatment was the same. However, the authors did not report the degree of degradation of the apple tissue. To our knowledge, no prior studies have examined the kinetics of lyophilisation with a specific degree of tissue degradation caused by PEF treatment. No studies were found to compare the kinetics of freeze drying preceded by vacuum freezing with the kinetics of freeze drying material frozen in air at different freezing rates.

Therefore, this study was undertaken to determine the effect of PEF on the kinetics of freeze drying of frozen material under conditions of low and high freezing rates. The freeze drying process integrated with vacuum freezing was also investigated. From a practical point of view, it was important to verify the hypothesis that the PEF pre-treatment will shorten the duration of the freeze drying process.

## 2. Materials and Methods

### 2.1. Material

The research material was Granny Smith apples. Fruits were purchased at the local market. The apples were stored in a refrigerator at 4–5 °C. The whole fruits were used in the PEF tests without removing the skin.

Apples without and after PEF treatment were cut into slices with a thickness of 10 mm using the CL50 slicer (Robot-Coupe, Vincennes, France).

### 2.2. PEF Treatment

The raw material (whole apples) was processed using a device manufactured by Elea GmbH (Model PEF Pilot™, Quakenbrück, Germany). The chamber of the device is equipped with stainless steel electrodes, the distance between the electrodes is 27 cm. The PEF device can generate an electric field with a voltage of up to 30 kV and an intensity of 180 A. In the experiment, the device was calibrated at 27 kV·cm^−1^, which gives the electric field strength *E* = 1 kV·cm^−1^. The apples were placed in a chamber filled with tap water at a temperature of 20 °C. The total mass of the sample and water was 1500 g. The pre-treatment of apples with a pulsed electric field (PEF) was carried out with a PEF Pilot ™ apparatus (Quakenbrück, Germany). During the PEF experiment, the system generated a voltage of 24 kV and pulses with a monopolar, exponential decay with a duration of 7 μs. The period between pulses was set to 0.05 s (20 Hz). The distance between the electrodes was 240 mm. The samples were placed in a chamber intended for PEF treatment and supplemented with water at room temperature to a total weight of 1500 g. The chamber was covered with a fitted Teflon cover, avoiding air pockets. After applying a specified number of pulses (from 0 to 400 pulses), the value of the set total energy, expressed in kJ, was noted. Whole apples were subjected to PEF treatment.

### 2.3. Freezing of Apples

The slices of apples (with and without PEF treatment) were frozen at different cooling rates in a shock freezer (Irinox, Corbanese, Italy). Fast freezing (FF) was carried out under forced convection conditions at an air temperature of −40 °C, at the maximum fan speed, for 3.5 h. Slow freezing (SF) was carried out in the same freezer, with a minimum fan operation, by programming a gradual decrease in the freezing temperature, according to the following sequence of parameters (air temperature and time): −4 °C/2 h, −7 °C/2 h, −10 °C/2 h, −12 °C/2 h, −15 °C/2 h and −40 °C/2 h. The vacuum-freezing process (VF) was carried out in a Gamma 1–16 freeze dryer chamber (Martin Christ Gefriertrocknungsanlagen GmbH, Osterode am Harz, Germany) while the chamber pressure was lowered to 63 Pa (freeze-drying pressure). The freeze dryer was connected to a Pfeiffer vacuum pump, model Duo 10 M (Nashua, NH, USA) with a capacity of 12 m^3^·h^−1^.

### 2.4. Freeze-Drying

Table 1 presents the kind of samples subjected to freeze drying. This process was carried out using a Gamma 1–16 freeze dryer (Martin Christ Gefriertrocknungsanlagen GmbH, Osterode am Harz, Germany). The pressure in the chamber was 63 Pa (corresponding to the ice temperature in thermodynamic equilibrium −25 °C). The set shelf temperature was 20 °C.

After the freeze-drying, the selected physical properties (water content, water activity, colour) of dried apples were investigated.

### 2.5. The Physical Properties of Apples and the Freeze-Dried Plant Material

#### 2.5.1. Electrical Conductivity Disintegration Index *Z* as a Measure of Cell Disintegration of Apple Tissue

The pH/conductivity meter CPC/401 (Elmetron, Zabrze, Poland) was used to measure the electrical conductivity of fresh and PEF-treated raw materials. Each measurement was repeated 5 times. Measurements were made on three different apples to take into account the diversity of the biological material. The cell disintegration index *Z* was determined using the formula [31]:

(1) $$Z=\frac{\left(\sigma -\sigma_{i}\right)}{\left(\sigma_{d}-\sigma_{i}\right)}$$

where *σ* is the value of measured electrical conductivity, the index *i* is conductivity after applying given energy, index *d* is conductivity of an apple after applying a dose of energy resulting in the complete destruction of apple tissue. On this basis, the relationship between the amount of applied specific energy, expressed in kJ·kg^−1^, and the electrical conductivity disintegration index *Z* was determined.

#### 2.5.2. Determination of the Water Content in the Freeze-Dried Material

Water content in the material after freeze-drying was determined using the vacuum drying method under pressure reduced to 2 kPa, at a temperature of 70 °C for 24 h, in a VO 200 vacuum dryer (Memmert GmbH, Büchenbach, Germany).

#### 2.5.3. Water Activity Measurement

The water activity was measured using a Hygrolab C laboratory analyser (Rotronic, Bassersdorf, Switzerland) with an accuracy of ±0.001.

#### 2.5.4. Colour Measurement

The colour parameters were analysed using a Konica-Minolta CM-5 chromameter with a measuring diaphragm diameter of 8 mm and a standard observer of 2°. The measurement was carried out using the reflection method in the CIE L*a*b* system with a standard D65 light source. Before the test, calibration was performed with the use of black and white standards. The measurement was carried out on samples after lyophilisation. The material was placed over the measurement gap in such a way as to measure the colour of the crumb, without the rind. The measurement was carried out in a minimum of 5 replicates for each sample. The total colour change was calculated for the sample that was not subjected to pulsed electric field treatment, fast-frozen and freeze-dried. The following formula was used:

(2) $$\Delta E=\sqrt{{\left(\Delta L^{*}\right)}^{2}+{\left(\Delta a^{*}\right)}^{2}{\left(\Delta b^{*}\right)}^{2}}$$

where: Δ*E*—total colour change, ΔL*—brightness difference between the tested sample and the control sample, Δa*—the difference in the a* coordinate value between the tested sample and the control sample, and Δb*—the difference in the b* coordinate value between the test sample and the control sample.

The browning index was determined as an indicator of the intensity of enzymatic and/or non-enzymatic browning reactions [32]. The *BI* was calculated using the following formula [33,34]:

(3) $$x=\frac{a^{*}+1.75L^{*}}{5.645L^{*}+a^{*}-3.012b^{*}\;}$$

(4) $$BI=\frac{100\left(x-0.31\right)}{0.17}$$

### 2.6. Kinetics of the Freeze-Drying of Apples and Mathematical Modelling of the Process

During the freeze-drying process, the weight loss of the lyophilised material was monitored online. A weight system model SWL025 (Mensor, Warsaw, Poland) was used. The measuring range was from 0.2 to 250 g with the resolution of 0.01 g. The measuring system was adapted to work in the temperature range from −20 to 70 °C. It was validated under vacuum conditions. The weighed sample was placed on an aluminium pan with a diameter of 15 cm, which during the process lay directly on the heating plate of the freeze dryer. The pan was lifted only at the moment of measuring the mass by means of a special mechanism. As a result, the weighing system was loaded only during the measurements, and the weighing did not disturb the temperature conditions of the freeze drying. The frequency of measurements for the first 120 min of the process was every 5 min, and then every 15 min.

On the basis of the weight loss during drying, the moisture ratio values *MR* (dimensionless) were calculated and drying curves (moisture ratio versus time) were plotted.

(5) $$MR=\frac{u-u_{e}}{u_{o}-u_{e}}$$

where: *u*—water content at the time, *u_o_*—initial water content, *u_e_*—equilibrium water content (g water·g^−1^ dry basis).

The selected mathematical models were fitted to the experimental data for the freeze-drying of apples (Table 2). Regression analysis was performed with the application of Table Curve v. 5.01 program (Systat Software Inc., Palo Alto, CA, USA).

The goodness of fit was evaluated by the determination coefficient (*R*^2^) and the root mean square error (*RMSE*).

(6) $$RMSE=\sqrt{\frac{\sum_{i=1}^{N}{\left(MR_{i,p}-MR_{i,e}\right)}^{2}}{N}}$$

where: *MR_i,p_*—the predicted moisture ratio, *MR_i,e_*—the experimental moisture ratio, *N*—number of experimental data.

The best model was used to calculate the drying rates as the first derivate *dMR*·*dt*^−1^ using the Table Curve v. 5.01 program (Systat Software Inc., Palo Alto, CA, USA). The drying rates curves were plotted: drying rate (min^−1^) versus moisture ratio (*MR*).

### 2.7. Statistical Methods

The Statistica v 13.3 (StatSoft Inc., Tulsa, OK, USA) program was used to perform the statistical analysis. One-way ANOVA analysis and paired Tukey’s Honest Significant Difference method test were used to evaluate the significance of the effect of freezing method and the number of applied pulses on the colour parameters as well as water content and water activity of dried samples. The data were presented as mean ± standard deviations.

## 3. Results and Discussion

### 3.1. Determination of the Dependence of on the Amount of Energy Supplied during Treatment with a Pulsed Electric Field on the Electrical Conductivity Disintegration Index

**Figure 1:**
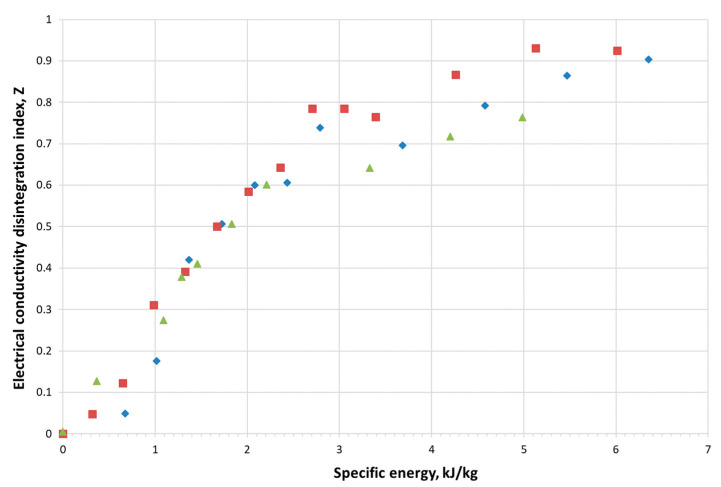
Relation between energy supplied during PEF treatment and the electrical conductivity disintegration index (various symbols and colours represent 3 different repetitions of the measurements).

The assumption of the research was to study the kinetics of the lyophilisation process for material with different degradation of cell membranes. To determine the energy levels applied to the material during PEF treatment prior to freeze drying, the material’s reaction to PEF was analysed. For this purpose, the relationship between the conductivity of apple tissue and different doses of PEF energy was determined. The theoretical basis of this relationship is the fact that the destruction of cell membranes causes the release of cell juice, which is a good current conductor. The more juice released from the cells, the higher the conductivity values of the conductometer should be. Further experiments were carried out on three different fruits, which also took into account the variability of the biological material. The obtained results are presented in Figure 1. The increase in the applied energy in the range from 0.3 to 3 kJ·kg^−1^ (approx. 50 to 500 pulses) increased the tissue damage index to 0.8. A linear increase in the tissue damage index *Z* was observed with increase in applied energy. A further increase in the energy used resulted in slight changes in the index. It means that after exceeding the dose of about 3 kJ·kg^−1^, cell juice filled the intercellular spaces, which may indicate the complete disintegration of cell membranes.

The highest, an abrupt increase in electrical conductivity disintegration index *Z* was observed in the range of 0.8—1.3 kJ·kg^−1^, which corresponded to the application of about 120 and 160 pulses, respectively. Therefore, the material was used for further research after applying such doses of specific energy. It is worth emphasizing that the presence of a cellular structure facilitates the sublimation of ice, as the pores and free spaces between cells create capillaries that facilitate the flow of mass (water vapor). The destruction of this structure hinders the sublimation process [28]. For this reason, it was decided to use such doses of PEF (120—160 pulses), which significantly increased the outflow of juice, but did not cause complete damage to the structure (electrical conductivity disintegration index *Z*, below 0.2 and about 0.5, respectively).

### 3.2. The Physical Properties of Apples and the Freeze-Dried Plant Material

For each experiment, the material was obtained with fairly low water content and water activity. The water content was from 2.81 to 4.25% d.m. The water activity was from 0.165 to 0.226 (Table 3). It was not found that the final water content or water activity depended on the technology used. However, the water content was higher than the non-freezing water content in the apple [41,42] and the water activity corresponded to the monolayer capacity [42]. It is advantageous, because for a water activity of 0.2 there is a minimum oxidation reactions rate [43], which is destructive for most biologically active compounds contained in food raw materials.

Colour parameters are a very important determinant of the quality of a food product and often determine the quality of the final product. The colour of foods most often has been measured in L*a*b*. This colour space is an international standard for colour measurements, adopted by the Commission Internationa‘ed’Eclairage (CIE) in 1976. Parameter L* represents brightness and has a range from 0 to 100. Parameter a* represents a degree of redness-greenish in the range from −120 to 120. A fresh apple darkens as a result of cutting (damaging the cells). The cause of the colour change of plant tissue is enzymatic browning reactions. The presence of the enzymes polyphenol oxidase (PPO) and polyphenols in apples leads to a browning reaction in contact with oxygen. This reaction also occurs in every case of apple cell disintegration, e.g., when the cells are crushed. There is a lot of data in the literature on the change in colour of apples during drying. The influence of the drying method (conventional, vacuum, microwave, freeze drying, and osmotic drying) on the colour parameters [44] as well as the effect of temperature and air relative humidity on changes in colour parameters during the drying of apple and other fruits [45] were found.

In the present studies, the highest value of the parameter L* = 71.54, statistically significantly different from the others, was found for samples without pre-treatment. However, the freezing method and the dose of energy during PEF treatment did not affect the value of this parameter. During slow and vacuum freezing, as well as in the material after PEF treatment, cell disintegration takes place and the release of both enzymes and polyphenols, which led to the enzymatic browning reaction. The values of a* parameter, for materials without PEF treatment ranged from −0.41 to 3.22, were significantly lower than the other materials, ranging from 4.24 to 7.82. The values of the b* parameter ranged from 18.09 to 27.11. These values were comparable to the values obtained by Kahraman et al. [46] for the freeze-dried apple without pre-treatment and lower compared to the values obtained for the ultrasonic-dried apples. For all materials, the obtained Δ*E* value is greater than 5, which means that the observer has the impression of two different colours. However, these values were also 30–50% lower compared to the literature values for convection-dried apples [46].

The browning index was the lowest for the material FF_0 (35.8) (Table 3). Slow freezing and vacuum freezing of both the material untreated with PEF caused a statistically significant increase in the *BI* index to values ranging from 51.1 to 60.8 with comparison to sample FF_0. These dependencies can be explained by the degree of cell damage. The application of 120 and 160 PEF pulses led to the oxidation reactions because a significant part of the cells was probably destroyed. During slow freezing, the large crystals formed which may lead the release of cell fluid during vacuum freezing. The intensive evaporation during pressure reduction can also cause cell disruption and release of browning reactants. Kahraman et al. [46] studied the colour of freeze-dried and ultrasound-dried apples. In these studies, the browning index was 48.71 for freeze-dried apples, which is a higher value than obtained for the FF_0 material. This may be due to different freezing conditions or a higher polyphenol content in the tested material. Application of ultrasound drying gave the value of *BI* equal to 64.6, so it was higher than the value observed in this study. Ultrasounds, PEF, slow freezing, or vacuum freezing, disturb the cell structure, which led to the intensification of browning processes. All values obtained in this study were significantly lower than the *BI* index for apples dried in hot air (91.54) [46]. Therefore, it can be concluded that although the PEF treatment induced browning reactions, the final material still had a lower *BI* index in comparison to the material obtained during convection drying.

### 3.3. Kinetics of the Freeze-Drying of Apples

**Figure 2:**
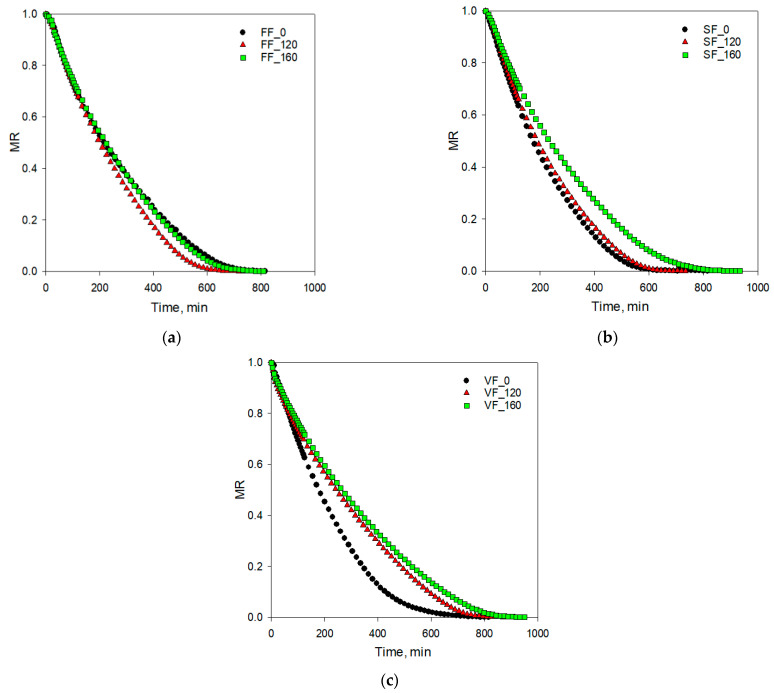
Experimental drying curves of apples with and without PEF treatment: (**a**) fast-frozen FF, (**b**) slow-frozen SF, (**c**) vacuum-frozen VF.

The course of the drying curves is shown in Figure 2. They are described using the models presented in Table 2. Table 4 shows the results of the regression analysis of the relation between moisture ratio and drying time. The goodness of fit of selected models to the experimental data was evaluated using the coefficients *R*^2^ and *RMSE*. The best model should be described by the highest values of determination coefficient *R*^2^ close to 1 and the root mean square error *RMSE* values should be close to 0 [47]. Most tested models were characterised by high values of determination coefficients from 0.981 to 0.999 with exception of model no 6 (two-term) (Table 4). The comparison of *RMSE* values showed that this statistical parameter gave values lower than 0.03 for models 2, 3, and 4. Among all investigated models, the model no 3 showed the lowest values of *RMSE* ranging from 0.0073 (for SF_120) to 0.0127 (for FF_120). Additionally, the determination coefficients for this model ranged from 0.998 to 0.999. The Mildilli et al. [38] model (no 3) represented the experimental data during the freeze drying of apples satisfactorily. Thus, this model was selected to characterise the drying curves of apples. Additionally, the Mildilli et al. [38] model was the most precise in describing the process of the convection drying of PEF-treated carrot [17] and the air-drying of a thin layer of Golden apples [48].

Table 5 presents the calculated values of the Midilli et al. model constants. The values of *a* and *n* constants did not differ for most dried samples. The highest value of drying coefficient *k* was observed for dried apples without PEF treatment and fast-frozen (2.6 × 10^−3^). Additionally, the high values of *k* constants (about 2 × 10^−3^) were noted for freeze-dried apples with PEF pre-treatment with a similar specific energy intake (120 pluses) fast and vacuum frozen. Gachovska et al. [49] reported that the application of PEF treatment increased the drying rate of air-dried carrots. However, the higher pulse number induced minimal effect on the drying rate and the degradation of carrots [49,50] and red bell pepper [51] plasmalemma. Results obtained by Wiktor et al. [52] showed that PEF treatment of apples increased *k* coefficients of the Midilli et al. model for most air-dried samples with the exception of apples treated by 50 pulses of PEF at electric field intensity 5 kV·cm^−1^ (value of *k* was similar as observed for intake apples). Other studies [17] did not report a clear relationship between the constants of model and PEF treatment conditions which is in agreement with our results.

Analysis of the experimental drying curves (Figure 2a–c) showed the different effects of PEF treatment in the case of applying the same freezing method of apples. The control samples (FF_0) as well as apples treated with PEF with 160 pulses which were frozen with a high rate had a similar course of drying curve (Figure 2a). Drying time (time required to obtain 0.05 water g·g^−1^ d.m.) of these samples ranged from 795 to 800 min. Application of 120 pulses during PEF treatment and fast-frozen method accelerated the freeze drying process (Figure 2a) and drying time was 720 min (Table 5). The opposite effect was observed for vacuum frozen apples (Figure 2c) using PEF pre-treatment with a higher number of pulses increasing the drying time from 840 to 935 min. The drying process was shortened to 785 min without application of PEF pre-treatment.

**Figure 3:**
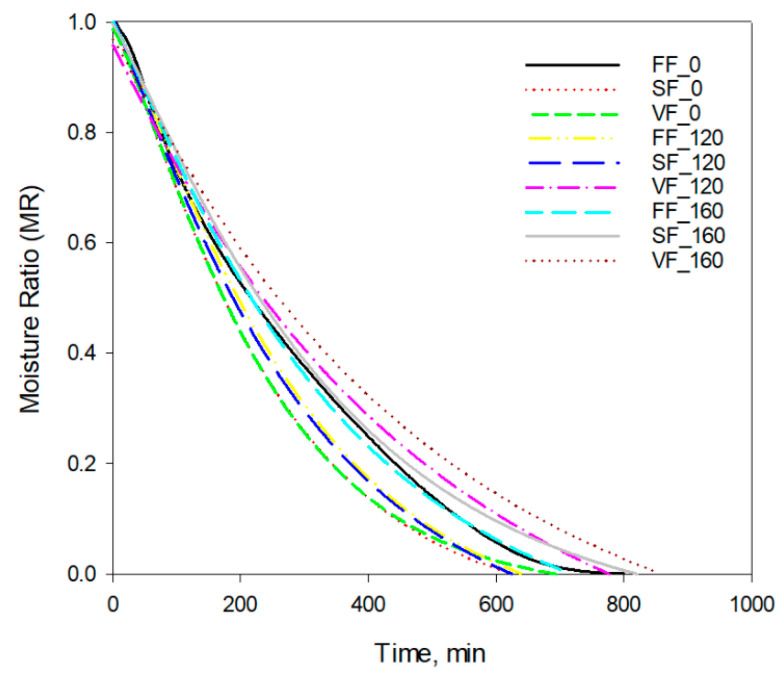
Drying curves predicted based on the Mildilli et al. model.

Our investigation indicates that effect of PEF treatment can be also related with method of freezing. Figure 3 presents the predicted drying curves obtained using the Midilli et al. model. The shortest drying process was observed for samples with application of slow freezing and 120 pulses of PEF. The rate of freezing affects the size of the formed crystals, which significantly affects the structure of the tissue. The larger ice crystals were formed and a higher disruption of fibrous tuna muscle was observed, that revealed by less integrity of fibrous structures [53].

It can be assumed that the combination of slow freezing and using the specific PEF treatment led to intensive electroporation and disintegration of apple cells. Vacuum freezing of samples with PEF pre-treatment (160 pulses) caused the significant extension of the duration of the drying process (Figure 3, Table 5). Lebovka et al. [54] compared the properties of air-dried potatoes obtained with PEF pre-treatment as well as using freezing, thawing of tissue before drying. The drying time was considerably reduced for freeze- thawed tissue. The PEF pre-treatment also allowed to enhance the drying process. However, the drying rates for PEF pre-treated samples did not exceed the values obtained for freeze-thawed potatoes. The authors explained this phenomenon of the different structure, density, porosity, and texture created as a result of application of thermal (freezing thawing) and non-thermal (PEF) processes. Some investigations emphasised that efficiency of PEF application before dehydration on the kinetics of drying depends on many factors (the value of electric field, frequency of electric field, number and width pulses, as well as process time) and it is very complex [25,55]. There are some optimal parameters of PEF treatment specific for plant tissue and other processing methods (e.g., dehydration) applied during the technological process [17,56].

The structure of the apple is porous. The increase in sublimation rate is probably related to the removal of water that freezes outside the cell, for which the resistance to mass transport is very low. Water removed from intercellular spaces causes evaporation of successive layers filling the pores. Thus, the evaporation area is increasing.

**Figure 4:**
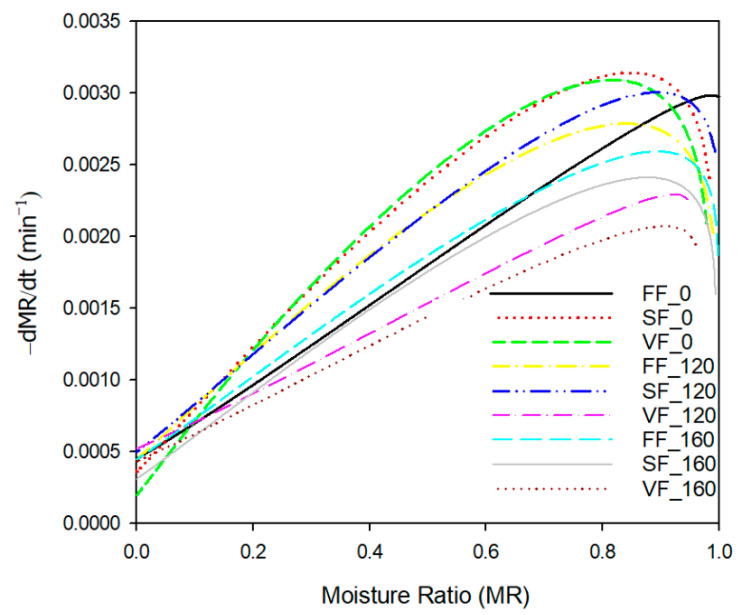
Drying rate curves of freeze-dried apples with different PEF treatment and freezing methods.

Figure 4 presents the drying rate curves obtained through the differentiation of Midilli et al. model [38]. At the beginning of the process the significant differences in values of maximum drying rate were observed. The lowest value of the maximum of the curve was observed at a moisture ratio of 0.9 and drying rate of 0.0021 min^−1^ for the PEF treated sample with 160 pulses and vacuum frozen. The maximum drying rate (0.0031 min^−1^ at *MR* = 0.86) was obtained for the intact sample frozen with a low rate. The drying rate at the beginning of the process was considerably high (0.0029 min^−1^) for the fast frozen control sample (FF_0). In this case, only air is present in the material (intercellular spaces), so the evaporation can also take place inside the pores. However, the rapid decrease in drying rate was observed after several minutes of the process. Application of PEF treatment resulted in smaller changes in drying rate during the whole process, e.g., the sample with PEF application (VF_160) reduced its drying rate at the end of the process about 5 times but a 15-fold decrease in drying rate was observed for the vacuum frozen sample without PEF treatment. Nowak et al. [28] observed that the freezing method (fast/slow) did not affect the sublimation time of celery tissue (during the freeze drying process). However, structure of material impacted the sublimation time. The pulp of celery had the longer sublimation time than untreated and blanched samples. The slow frozen pulp sublimated with a longer period than the fast frozen sample. The quick-frozen solutions (with small crystals) could dry slowly due to formation of small pores with higher resistance to the mass transfer. It may limit the sublimation rate. The extension of freeze drying time can be related to the smaller evaporation surface of non-porous samples with comparison to material with non-destroyed cell structures. Formation of crust layers on the apple tissue surface also could limited vapor diffusion and sublimation processes [57]. The degree of structure destruction can affect the behaviour of the material during the freezing and the freeze drying. This can also be observed for materials after PEF treatment. Destruction of the cell structure (cell membranes) in 50% (160 pulses) resulted in filling of the intercellular spaces with the cellular fluid and the loss of the positive effect related to the porosity of the material.

The application of PEF and different freezing methods may accelerate the intensity changes in tissue structures (mainly electroperforation of the cell membranes). It is a reason why most PEF-treated samples had a lower drying rate at the beginning of the process when sublimation occurs. At the second stage of freeze drying (desorption drying), the drying rates of PEF-treated samples decreased slower than the control samples (without PEF treatment). It may be assumed that the damage of tissue caused by PEF treatment affected the drying rate to a lesser degree at the final drying stage when the material had porous structure and low content of free water.

## 4. Conclusions

Based on the performed research, it can be concluded that the degree of cell damage, which was the result of pre-treatment with PEF and/or the size of the crystals, depending on the freezing rate affected the kinetics of the lyophilisation process. PEF pre-treatment caused destruction of cells at the level of 25% (electrical conductivity disintegration index *Z* = 0.25). Thus, application of PEF treatment and slow or quick freezing had a positive effect on reduction in the freeze drying time. However for samples without PEF treatment, application of slow freezing caused a decrease in freeze drying time by about 10% in comparison to fast frozen materials (base technology—FF_0). These technologies can be recommended for optimization of the apple freeze drying process. More intensive destruction of the cellular structure caused by PEF treatment damage 50% of cells, and, additionally, slow freezing or vacuum freezing resulted in the negative effect of increase in the drying time by about 15%. Summing up, it can be concluded that it was possible to obtain a positive effect of PEF treatment on the shortening of drying time. However, the appropriate degree of destruction of the cellular structure should be selected. The hypothesis was confirmed. The PEF processing of apple tissue, as well as modifications of the freezing process in relation to the base technology, cause a significant change in colour parameters and an increase in the browning index (*BI*). This should be taken into account when selecting the technology to obtain the required properties of the final product.

## Figures and Tables

**Table 1:** Description of the freeze-dried samples.

Samples	Number of Pulses (PEF Treatment)	Supplied Specify Energy (kJ/kg)	Method of Freezing
FF_0	0	0	Fast freezing
SF_0	0	0	Slow freezing
VF_0	0	0	Vacuum freezing
FF_120	120	896.3	Fast freezing
SF_120	120	941.4	Slow freezing
VF_120	120	928.9	Vacuum freezing
FF_160	160	1361.5	Fast freezing
SF_160	160	1306.2	Slow freezing
VF_160	160	1326.8	Vacuum freezing

**Table 2:** Mathematical models applied to the drying process.

Number of Model	Name of Model	Equation of Model	Reference
1	Newton	MR=exp(−k·t)	[35,36]
2	Logistic	MR=b/(1+exp(k·t)	[37]
3	Midilli et al.	$MR=a\cdot \exp\left(-k\cdot t^{n}\right)+b\cdot t$	[38]
4	Logarithmic	MR=a·exp(−k·t)+b	[36,39]
5	Henderson and Pabis	MR=a·exp(−k·t)	[40]
6	Two terms	$MR=a\cdot \exp\left(-k\cdot t\right)+b\cdot \exp\left(-k_{i}\cdot t\right)$	[36,39]

**Table 3:** The physical attributes of the freeze-dried apples obtained with different pre-treatment and freezing methods.

Samples	Water Content, %	Water Activity	L*	a*	b*	Δ*E*	*BI*
FF_0	3.55 ± 0.07 ^c^ *	0.226 ± 0.004 ^a^	71.54 ± 0.21 ^a^	−0.41 ± 0.20 ^d^	22.25 ± 0.36 ^d^	(—) **	35.8 ± 1.0 ^b^
SF_0	3.53 ± 0.11 ^c^	0.209 ± 0.001 ^c^	63.93 ± 0.24 ^b^	3.10 ± 0.68 ^c^	27.11 ± 0.32 ^a^	9.07 ± 0.31 ^c^	57.2 ± 0.3 ^a^
VF_0	2.99 ± 0.16 ^d^	0.165 ± 0.001 ^f^	63.28 ± 0.47 ^bc^	3.22 ± 0.57 ^c^	24.34 ± 1.06 ^bc^	9.31 ± 0.46 ^c^	53.1 ± 3.0 ^a^
FF_120	3.77 ± 0.01 ^b^	0.217 ± 0.002 ^b^	61.32 ± 1.06 ^bc^	7.20 ± 1.03 ^ab^	24.66 ± 0.11 ^bc^	12.97 ± 1.45 ^a^	59.0 ± 2.3 ^a^
SF_120	2.81 ± 0.08 ^d^	0.192 ± 0.003 ^e^	62.87 ± 2.78 ^b^	5.83 ± 1.65 ^b^	24.02 ± 0.84 ^bc^	10.83 ± 3.31 ^abc^	54.4 ± 7.5 ^a^
VF_120	3.61 ± 0.07 ^c^	0.202 ± 0.002 ^d^	63.59 ± 0.53 ^b^	6.65 ± 0.65 ^ab^	23.85 ± 0.62 ^bcd^	10.77 ± 0.78 ^b^	53.8 ± 2.6 ^a^
FF_160	3.61 ± 0.08 ^c^	0.188 ± 0.002 ^e^	63.99 ± 1.96 ^b^	6.30 ± 0.94 ^ab^	25.01 ± 0.53 ^b^	10.51 ± 2.00 ^bc^	55.9 ± 4.0 ^a^
SF_160	4.25 ± 0.06 ^a^	0.211 ± 0.008 ^bc^	59.09 ± 4.29 ^c^	7.82 ± 1.65 ^a^	23.63 ± 1.25 ^d^	15.03 ± 4.59 ^a^	60.8 ± 11.5 ^a^
VF_160	3.59 ± 0.01 ^c^	0.226 ± 0.001 ^a^	68.37 ± 1.77 ^a^	4.24 ± 0.92 ^c^	18.09 ± 1.17 ^e^	7.31 ± 0.77 ^d^	36.0 ± 3.1 ^b^

* the different letters in the columns indicate the significant difference between the parameters, *p* ≤ 0.05; ** total colour change Δ*E* was calculated with the reference to control sample (FF_0).

**Table 4:** The statistical results (*R*^2^, *RMSE*) of fitting to drying models (1–6) for different freeze-dried apples.

No. Model	Types of Freeze-Dried Apples								
	FF_0	SF_0	VF_0	FF_120	SF_120	VF_120	FF_160	SF_160	VF_160
1	0.0439 *	*0.0460*	*0.0430*	*0.0547*	*0.0477*	*0.0418*	*0.0484*	*0.0436*	*0.0404*
	(0.984) **	(0.984)	(0.985)	(0.976)	(0.981)	(0.984)	(0.981)	(0.987)	(0.985)
2	*0.0109*	*0.0187*	*0.0145*	*0.0247*	*0.0198*	*0.0270*	*0.0235*	*0.0198*	*0.0239*
	(0.996)	(0.996)	(0.993)	(0.995)	(0.996)	(0.993)	(0.995)	(0.997)	(0.994)
3	*0.0113*	*0.0101*	*0.0101*	*0.0127*	*0.0073*	*0.0133*	*0.0123*	*0.0114*	*0.0103*
	(0.999)	(0.999)	(0.999)	(0.998)	(0.999)	(0.998)	(0.998)	(0.999)	(0.999)
4	*0.0109*	*0.0141*	*0.0181*	*0.0160*	*0.0093*	*0.0124*	*0.0129*	*0.0129*	*0.0104*
	(0.999)	(0.998)	(0.997)	(0.998)	(0.998)	(0.998)	(0.998)	(0.998)	(0.999)
5	*0.0353*	*0.0371*	*0.0337*	*0.0434*	*0.0375*	*0.0420*	*0.040*	*0.0360*	*0.0395*
	(0.990)	(0.989)	(0.991)	(0.985)	(0.988)	(0.984)	(0.987)	(0.989)	(0.986)
6	*0.0420*	*0.3580*	*0.0930*	*0.0730*	*0.0779*	*0.0511*	*0.481*	*0.0369*	*0.0505*
	(0.985)	(0.871)	(0.935)	(0.956)	(0.952)	(0.977)	(0.982)	(0.989)	(0.978)

* Italicized values present *RMSE*; ** values in brackets present *R*^2^.

**Table 5:** Drying time and *a*, *b*, *k*, *n* constants of the Midilli et al. model for freeze-dried apples.

Samples	*a*	10^−5^ × *b*	10^−3^ × *k*	*n*	Drying Time, Min
FF_0	1.020 (0.001) *	−13.0 (1.2)	2.6 (0.3)	1.031 (0.020)	800
SF_0	0.994 (0.004)	−7.9 (0.8)	1.5 (0.2)	1.180 (0.018)	720
VF_0	0.987 (0.005)	−3.6 (0.6)	1.2 (0.2)	1.232 (0.017)	785
FF_120	0.998 (0.059	−10.0 (1.2)	1.1 (0.2)	1.195(0.024)	720
SF_120	1.000 (0.004)	−10.0 (0.9)	1.8 (0.1)	1.124 (0.014)	705
VF_120	0.958 (0.006)	−20.1 (1.6)	2.0 (0.3)	1.035 (0.028)	840
FF_160	1.001 (0.006)	−10.0 (1.1)	1.6 (0.2)	1.119 (0.023)	795
SF_160	0.995 (0.005)	−7.47 (0.8)	1.3 (0.1)	1.138 (0.019)	920
VF_160	0.969 (0.004)	−13.0 (0.9)	1.6 (0.2)	1.066 (0.020)	935

* Standard error values are in brackets.

## Data Availability

The data generated or analysed during this study are available from the corresponding author on reasonable request.
